# Barriers and Facilitators to Healthy Lifestyle Changes in Minority Ethnic Populations in the UK: a Narrative Review

**DOI:** 10.1007/s40615-016-0316-y

**Published:** 2016-12-07

**Authors:** Naina Patel, Harriet Batista Ferrer, Freya Tyrer, Paula Wray, Azhar Farooqi, Melanie J. Davies, Kamlesh Khunti

**Affiliations:** 1 Diabetes Research Centre, University of Leicester, Leicester, UK; 2 School of Social and Community Medicine, University of Bristol, Bristol, UK; 3 Department of Health Sciences, University of Leicester, Leicester, UK; 4 Leicester City Clinical Commissioning Group, Leicester, UK

**Keywords:** Diabetes, Ethnic minority populations, South Asian, UK, Healthy lifestyle changes, Narrative review

## Abstract

Minority ethnic populations experience a disproportionate burden of health inequalities compared with the rest of the population, including an increased risk of type 2 diabetes (T2DM). The purpose of this narrative review was to explore knowledge and attitudes around diabetes, physical activity and diet and identify barriers and facilitators to healthy lifestyle changes in minority ethnic populations in the UK. The narrative review focused on three key research topics in relation to barriers and facilitators to healthy lifestyle changes in minority adult ethnic populations: (i) knowledge and attitudes about diabetes risk; (ii) current behaviours and knowledge about physical activity and diet; and (iii) barriers and facilitators to living a healthier lifestyle. Nearly all of the studies that we identified reported on South Asian minority ethnic populations; we found very few studies on other minority ethnic populations. Among South Asian communities, there was generally a good understanding of diabetes and its associated risk factors. However, knowledge about the levels of physical activity required to gain health benefits was relatively poor and eating patterns varied. Barriers to healthy lifestyle changes identified included language barriers, prioritising work over physical activity to provide for the family, cultural barriers with regard to serving and eating traditional food, different perceptions of a healthy body weight and fear of racial harassment or abuse when exercising. Additional barriers for South Asian women included expectations to remain in the home, fear for personal safety, lack of same gender venues and concerns over the acceptability of wearing ‘western’ exercise clothing. Facilitators included concern that weight gain might compromise family/carer responsibilities, desire to be healthy, T2DM diagnosis and exercise classes held in ‘safe’ environments such as places of worship. Our findings suggest that South Asian communities are less likely to engage in physical activity than White populations and highlight the need for health promotion strategies to engage people in these communities. There is a gap in knowledge with regard to diabetes, physical activity, diet and barriers to healthy lifestyle changes among other ethnic minority populations in the UK; we recommend further research in this area.

## Background

Minority ethnic populations experience a disproportionate burden of health inequalities in a number of disease areas compared with the rest of the population. These include an increased prevalence of type 2 diabetes mellitus (T2DM), reported to be up to six times higher among South Asian (Indian, Pakistani, Bangladeshi and Sri Lankan) communities [1–5] and up to three times higher among Black African and Black Caribbean communities [6] compared with White populations in the UK.

Increasing levels of obesity and sedentary lifestyles have been associated with a rise in T2DM [7, 8]. However, the relationship between obesity and ethnicity is not always clear. On the surface, it would appear that ethnic minority groups (at least men) have a lower risk of obesity compared with the White population. The 2004 Health Survey for England reported that the prevalence of obesity was lower among men from Black African, Indian, Pakistani, Bangladeshi and Chinese minority ethnic groups. In contrast, women from Black African, Black Caribbean and Pakistani communities (but not Chinese) had higher rates of obesity [9]. However, it is argued that, in South Asian communities, a substantially lower body mass index (BMI) and waist circumference are needed to confer equivalent risk factor profiles [10, 11] because a more centralised distribution of body fat is typically observed [12, 13]. This has led to the World Health Organisation (WHO) recommending a lower BMI threshold for obesity (27.5 kg/m^2^) for South Asian populations [14], and this threshold is lower still in the UK (25.0 kg/m^2^) [15]. The new threshold would indicate higher rates of obesity among Indian, Bangladeshi men and women and higher rates of obesity among Pakistani women compared with White populations [16]. The Chinese population has also been identified as having higher blood pressure level at significantly lower BMI values compared with White Europeans [10, 17], but BMI thresholds are yet to be agreed.

Global and national guidance recognise the importance of the prevention of chronic diseases. The UK follows WHO guidance, which recommends limiting energy intake from total fats, replacing saturated fats with unsaturated fats, increasing intake of fruit and vegetables, whole grains and nuts and limiting simple sugars, salt and sodium [7, 18]. However, it is acknowledged that dietary habits vary within and between ethnic groups and are influenced by multiple factors, including food availability, financial resources, health, food and religious beliefs and cultural customs [19].

The importance of physical activity is also recognised in national and international guidance. For adults, at least 150 min of moderate intensity or 75 min of vigorous intensity physical activity each week is recommended [20–22]. Currently, recommendations do not differ by ethnic group but the influence of heritability on cardiovascular fitness is well established [23, 24]. There is increasing evidence of a relationship between ethnicity, physical activity, risk factors for metabolic disease and the amount of physical activity required to achieve low cardio-metabolic disease risk [25–27]. However, much of the variability in cardio-respiratory fitness appears to be due to non-genetic factors [23, 24, 28].

Lifestyle changes play a key role in preventing or delaying the development of T2DM. Results from large, clinical trials demonstrate that relatively modest changes in diet and physical activity can reduce the development of T2DM by around half [29, 30]. Systematic review evidence suggests that ‘real-world’ lifestyle interventions are both effective [31, 32] and cost-effective [33]. However, to maximise the effectiveness of these interventions in minority ethnic groups, the barriers and facilitators to healthy lifestyle changes need to be identified and understood, so that health disparities can ultimately be reduced.

In this narrative review, we explore knowledge and attitudes about diabetes risk, physical activity and diet and identify barriers and facilitators to healthy lifestyle changes in minority ethnic populations in the UK. This work formed part of a longer term strategy to inform the development and implementation of a social marketing campaign to be conducted in Leicester, UK.

## Methods

### Search Strategy and Selection Criteria

For this study, we conducted a narrative review, focusing on three key research topics in relation to barriers and facilitators to healthy lifestyle changes in minority ethnic populations in the UK: (i) knowledge and attitudes about diabetes risk; (ii) current behaviours and knowledge about physical activity and diet; and (iii) barriers and facilitators to living a healthier lifestyle. We used an emergent (‘berry picking’) model of information retrieval [34], starting with a general query on the key research topics and using both ‘backward chaining’ (moving backwards through a chain of reference lists) and ‘forward chaining’ (following a chain of citations in a forward direction) to identify primary research studies.

Inclusion criteria were primary studies involving adult minority ethnic groups residing in the UK. Studies restricted to children and adolescents were not included. We did not restrict to study type but focused on study designs that focused on interventions around T2DM in Black and minority ethnic communities/populations. We included studies reporting on the perspectives of participants with and without T2DM as some of the issues affecting healthy behaviour change, such as social norms and values, are likely to be equally applicable to both groups. Where the issues appeared to differ, the diabetes status of the participants was clarified.

For each article we extracted author, year of publication, setting, data collection methods and patient characteristics.

## Results

We identified 34 articles or reports relevant to knowledge and attitudes about diabetes risk; current behaviours, knowledge and attitudes about physical activity and diet; and barriers and facilitators to living a healthier lifestyle [9, 35–67]. The articles retrieved are summarised in Table 1. Most of the studies used qualitative methods in the form of focus groups or interviews. Almost all described South Asian minority ethnic populations, either as a group or restricted to Indian, Pakistani or Bangladeshi communities [9, 36–55, 58–67]. Seven of these studies also included other ethnic minority populations [9, 36, 43, 48, 54, 60, 61]; the remaining study focused on the Somali community only [56]. Seven of the studies were restricted to people with diabetes [42, 43, 51–53, 62, 63]; five of these referred or restricted to people with T2DM [51–53, 62, 63]

**Table 1:** Characteristics of studies in the review of minority ethnic minority populations in the UK

Study	Theme	Setting	Objectives of study	Design	Participants
Williams et al. [65]	Current behaviours, knowledge and attitudes about physical activity and diet	Glasgow, Scotland	To develop a profile of non-biochemical coronary risks for the South Asian population and the general population in Glasgow, with a focus on dietary patterns and potential causes of stress	Questionnaire	Number (% male), not clear (NR). Mean age (range) in years, 35 (30–40). Ethnic categories: South Asian (89% from India subcontinent) and general population
Wyke and Landman [66]	Current behaviours, knowledge and attitudes about physical activity and diet	Glasgow, Edinburgh and Stirling, Scotland	To explore diet and cuisine among family members from a range of South Asian origins	Focus groups and semi-structured interviews	Number (% male), 93 (34%). Mean age (range) in years, NR (NR). Ethnic categories: South Asian (Bangladeshi, Pakistani and Indian)
Rai and Finch [60]	Barriers and facilitators to living a healthier lifestyle	England	To investigate attitudes towards, and barriers to, physical activity among South Asian and Black communities in England	Focus groups	Number (% male), 175 (50%). Mean age (range) in years, NR (18–50). Ethnic categories: South Asian (Indian, Pakistani and Bangladeshi; *n* = 109; 49% male) and Black (African and Caribbean; *n* = 66; 52% male)
Bush et al. [36]	Barriers and facilitators to living a healthier lifestyle	Glasgow, Scotland	To explore family hospitality and ethnic tradition among South Asian, Italian and general population women	Structured interviews	Number (% male), 259 (all women). Mean age (range) in years, 30 (20–40). Ethnic categories: Italian (*n* = 90), South Asian (*n* = 119) and general population (*n* = 50)
Greenhalgh et al. [43]	Knowledge and attitudes about diabetes risk	London, England	To explore the experience of diabetes in British Bangladeshis	Narratives, focus groups, interviews, pile sorting exercises	Number (% male), 50 (NR). Mean age (range) in years, NR. Ethnic categories: South Asian (Bangladeshi; *n* = 40), White (*n* = 8) and Black (African/Caribbean; *n* = 2). Other restrictions: diabetes only
	Current behaviours, knowledge and attitudes about physical activity and diet				
	Barriers and facilitators to living a healthier lifestyle				
Jamal [46]	Barriers and facilitators to living a healthier lifestyle	Bradford, England	To explore food consumption experiences of British Pakistanis	Interviews and participant observation	Number (% male), 37 (‘mostly’ male). Mean age (range) in years, NR. Ethnic categories: South Asian (Pakistani)
Farooqi et al. [38]	Knowledge and attitudes about diabetes risk	Leicester, England	To identify key issues relating to attitudes and knowledge of lifestyle risk factors for coronary heart disease among South Asians aged over 40 years	Focus groups	Number (% male), 44 (55%). Mean age (range) in years, 54 (40+). Ethnic categories: South Asian (all)
	Current behaviours, knowledge and attitudes about physical activity and diet				
	Barriers and facilitators to living a healthier lifestyle				
Johnson et al. [48]	Current behaviours, knowledge and attitudes about physical activity and diet	England	To report on issues relevant to circulatory disorders (including diabetes) in ethnic minority populations. To describe characteristics of people who make up Black and minority ethnic group communities	Second national survey of Black and minority ethnic groups conducted in 1994	Number (% male), 4452 (weighted sample by age and gender). Ethnic categories: South Asian (Indian/East African (*n* = 1608), Pakistani (*n* = 1552) and Bangladeshi (*n* = 1533) and Black (African, Caribbean; *n* = 1990)
Johnson [49]	Barriers and facilitators to living a healthier lifestyle	England (survey). England (focus group study). Birmingham (interview study)	To identify barriers to healthy physical activity among Asian communities	Two ‘Health and Lifestyle’ surveys (1992 and 1994), focus group study [60] and social action research interviews [76]	Number (% male): Survey, not clear; focus groups, 109 (49%); and interviews, 377 (all men). Mean age (range) in years: survey, 16–74; focus groups, 18–50; and interviews, NR. Ethnic categories: Asian (Indian, Pakistani, Bangladeshi, Sri Lankan, Arabic and Chinese)
Patel et al. [58]	Barriers and facilitators to living a healthier lifestyle	Newcastle, England	To compare self-perception of body weight in South Asian and European women	Questionnaire and interviews, anthropometric measures and blood glucose	Number (% male), 770 (all women). Mean age (range) in years, NR (25–74). Ethnic categories: South Asian (Indian, Pakistani and Bangladeshi; *n* = 371) and European (*n* = 399), *N* = 770 (all women)
Carroll et al. [37]	Current behaviours, knowledge and attitudes about physical activity and diet	Bradford, Leicester, East Lancashire and Birmingham, England	To undertake case studies of ‘exercise on prescription’ schemes where provision is made for South Asian Muslim women	National survey of general practises and leisure centres and in-depth interviews	Number (% male), 35 (all women). Mean age (range) in years, NR (‘varied’). Ethnic categories: South Asian (Pakistani and Bangladeshi). Additional restrictions: all women on ‘exercise on prescription’ schemes
	Barriers and facilitators to living a healthier lifestyle				
Fischbacher et al. [39]	Current behaviours, knowledge and attitudes about physical activity and diet	UK	Systematic review to assess the evidence that physical activity is lower in South Asian groups than in the general population	Systematic review	Number of studies, 12 in adults; 5 in children. Ethnic categories: South Asian (Indian, Pakistani and Bangladeshi) and general population
Anderson et al. [35]	Current behaviours, knowledge and attitudes about physical activity and diet	Glasgow, Scotland	To identify differences in the evolution of the diets of South Asian and Italian migrants	Structured interviews	Number (% male), 175 (all women). Mean age (range) in years, 30 (30–40). Ethnic categories: South Asian migrants (*n* = 35), British South Asians (*n* = 37), Italian migrants (*n* = 30), British Italians (*n* = 38) and general population (*n* = 35)
Greenhalgh et al. [42]	Knowledge and attitudes about diabetes risk	London, England	To explore body image perception in British Bangladeshis with diabetes	Interviews (survey)	Number (% male), 96 (51%). Mean age (range) in years, 52 (NR). Ethnic categories: South Asian (Bangladeshi). Additional restrictions: diabetes only
	Current behaviours, knowledge and attitudes about physical activity and diet				
	Barriers and facilitators to living a healthier lifestyle				
Heald et al. [44]	Current behaviours, knowledge and attitudes about physical activity and diet	Sandwell, England	To determine the effects of total energy intake on the insulin-like growth factor system in two populations with markedly different macronutrient intake and cardiovascular event rate	Food diaries, anthropometric measures, blood tests, physical activity monitors	Number (% male), 536 (48%); *n* = 451 completed blood sample only. Mean age (range) in years, 49 (≥25 years). Ethnic categories: South Asian (Indian UK migrants; *n* = 242) and South Asian (Indian non-migrants; *n* = 294)
Stone et al. [63]	Knowledge and attitudes about diabetes risk	Leicester, England	To explore the experience and attitudes of primary care patients with diabetes living in a community with a high proportion of South Asian patients of Indian origin, with particular reference to patient empowerment	Semi-structured interviews	Number (% male), 20 (45%). Mean age (range) in years, 57 (33–80). Ethnic categories: South Asian (Indian; *n* = 15) and White (*n* = 5). Additional restrictions: all diabetes (*n* = 18 T2DM)
	Current behaviours, knowledge and attitudes about physical activity and diet				
	Barriers and facilitators to living a healthier lifestyle				
Lawton et al. [52]	Knowledge and attitudes about diabetes risk	Edinburgh, Scotland	To explore patients’ perceptions and experiences of undertaking physical activity as part of their diabetes care	Interviews	Number (% male), 32 (47%). Mean age (range) in years, 59 (≥30 years). Ethnic categories: South Asian (Pakistani; *n* = 23) and Indian; *n* = 9). Additional restrictions: all T2DM
	Current behaviours, knowledge and attitudes about physical activity and diet				
	Barriers and facilitators to living a healthier lifestyle				
Sproston and Mindell [9]	Current behaviours, knowledge and attitudes about physical activity and diet	England	England-wide health survey to monitor trends, estimate prevalence of health conditions and risk factors for specified health conditions. Reporting includes differences between subgroups of the population.	Interviews (survey)	Number (% male), 17,199 (8077 adults, 2003 children). Weighted sample. Ethnic categories: White, South Asian (Indian, Pakistani and Bangladeshi) and Black African/Caribbean (Chinese)
Lawton et al. [53]	Knowledge and attitudes about diabetes risk	Edinburgh, Scotland	To explore perceptions and understandings of T2DM causation, focusing on responsibility and blame for developing the disease	In-depth interviews	Number (% male), 53 (47%). Mean age (range) in years, 57 (33–78). Ethnic categories: South Asian (Pakistani (*n* = 23) and Indian (*n* = 9)) and White (*n* = 32). Additional restrictions: all T2DM
Netto et al. [57]	Knowledge and attitudes about diabetes risk	Edinburgh, Scotland	To consider how service user perspectives can be used to develop effective culturally focused coronary heart disease prevention interventions in Bangladeshi, Indian and Pakistani communities by addressing identified barriers, including deeply held cultural beliefs	Focus groups	Number (% male), 55 (44%; 1st focus group only as majority were interviewed twice). Mean age (range) in years, NR (≥16 years). Ethnic categories: South Asian (Indian (*n* = 20), Pakistani (*n* = 15) and Bangladeshi (*n* = 20))
	Current behaviours, knowledge and attitudes about physical activity and diet				
	Barriers and facilitators to living a healthier lifestyle				
Sriskantharajah and Kai [62]	Current behaviours, knowledge and attitudes about physical activity and diet	UK	To explore influences on, and attitudes towards, physical activity among South Asian women with chronic heart disease and diabetes to inform secondary prevention strategies.	Interviews	Number (% male), 15 (all women). Mean age (range) in years, 52 (26–70). Ethnic categories: South Asian (Indian, Pakistani, Bangladeshi, East African Asian and Sri Lankan). Additional restrictions: all coronary heart disease (*n* = 9) and/or T2DM (*n* = 8)
	Barriers and facilitators to living a healthier lifestyle				
Fleming et al. [40]	Barriers and facilitators to living a healthier lifestyle	Northwest England	To explore the influence of culture on T2DM self-management in Gujurati Muslim men	Interview and participant observation	Number (% male), 5 (all men). Mean age (range) in years, NR (52–72). Ethnic categories: South Asian (Indian/East Africa)
Grace et al. [41]	Knowledge and attitudes about diabetes risk	London, England	To understand lay beliefs and attitudes, religious teachings and professional perceptions in relation to diabetes prevention in the Bangladeshi community	Focus groups and semi-Structured interviews	Number (% male), 80 (46%; participants also included 20 religious leaders and Islamic scholars and 28 health professionals). Mean age (range) in years, 35 (NR). Ethnic categories: South Asian (Bangladeshi). Additional restrictions: no diabetes
	Current behaviours, knowledge and attitudes about physical activity and diet - Barriers and facilitators to living a healthier lifestyle				
Khanam and Costarelli [50]	Knowledge and attitudes about diabetes risk	London, England	To investigate the attitudes and beliefs held by UK Bangladeshi women on health and exercise and explore possible ways of increasing levels of physical activity in this group	Interview-guided questionnaire	Number (% male), 25 (women only). Mean age (range) in years, 47 (30–60). Ethnic categories: South Asian (Bangladeshi)
	Current behaviours, knowledge and attitudes about physical activity and diet				
	Barriers and facilitators to living a healthier lifestyle				
Lawton et al. [51]	Barriers and facilitators to living a healthier lifestyle	Edinburgh, Scotland	To explore food and eating practises from the perspectives of Pakistanis and Indians with type 2 diabetes, their perceptions of the barriers and facilitators to dietary change, and the social and cultural factors informing their accounts	Interviews	Number (% male), 32 (47%). Mean age (range) in years, ‘most’ in 50s and 60s (33–71). Ethnic categories: South Asian (Indian (*n* = 9) and Pakistani (*n* = 23)). Additional restrictions: all T2DM
Long et al. [54]	Current behaviours, knowledge and attitudes about physical activity and diet	UK	A systematic review of the literature on participation in sport and recreation by Black and minority ethnic communities. Includes analysis of the ‘Active People’ survey (Tier 3).	Systematic review	Ethnic categories: White (British, Irish and other), Asian (Indian, Pakistani, Bangladeshi, other), Black (African, Caribbean and other), mixed, Chinese and other
McEwen et al. [56]	Current behaviours, knowledge and attitudes about physical activity and diet	London, England	To understand dietary beliefs and eating behaviours of Somalis in the UK	Focus groups and a questionnaire survey	Number (% male), 139 (at least 67%). Mean age (range) in years, NR (NR). Ethnic categories: Somali
Scottish Ethnicity and Health Research Survey Working Group [61]	Current behaviours, knowledge and attitudes about physical activity and diet	Scotland	To review studies on ethnicity and health in Scotland	Review	Ethnic categories: White (Scottish, Irish and other), South Asian (Indian, Pakistani, Bangladeshi and East African), Black (Caribbean, African), Italian, Chinese and Scottish travellers
Yates et al. [67]	Current behaviours, knowledge and attitudes about physical activity and diet	Leicester, England	To investigate levels of physical activity and their association with health in a White European and South Asian population	Interviews, anthropometric measures	Number (% male), 5474 (48%). Mean age (range) in years, 56 (25–75). Ethnic categories: South Asian (Indian, Pakistani, Bangladeshi and other (*n* = 1164)) and White (*n* = 4310)
Ludwig et al. [55]	Knowledge and attitudes about diabetes risk	Greater Manchester, England	To explore health perceptions, diet and the social construction of obesity and how this relates to the initiation and maintenance of a healthier diet in UK Pakistani women	Focus groups and interviews	Number (% male), 55 (women only). Median age (range) in years, 45 (23–80). Ethnic categories: South Asian (Pakistani)
	Current behaviours, knowledge and attitudes about physical activity and diet				
	Barriers and facilitators to living a healthier lifestyle				
Williams et al. [64]	Current behaviours, knowledge and attitudes about physical activity and diet	England	To compare physical activity levels between South Asians and UK White population	Use of interview data from the Health Survey for England (1999–2004)	Number (% male), 14,395 (45%). Mean age (range) in years, 37 (≥16 years). Ethnic categories: South Asian (*n* = 5421) and White (*n* = 8974)
Jepson et al. [47]	Current behaviours, knowledge and attitudes about physical activity and diet	Aberdeen, Glasgow and Edinburgh, Scotland	To explore the motivating and facilitating factors likely to increase physical activity for South Asian adults and their families, in order to develop successful interventions and services	Focus groups and semi-structured interviews	Number (% male), 59 (~40%). Mean age (range) in years, NR (Adults). Ethnic categories: South Asian (Indian (*n* = 36), Pakistani (*n* = 17) and Bangladeshi (*n* = 6))
	Barriers and facilitators to living a healthier lifestyle				
Horne et al. [45]	Current behaviours, knowledge and attitudes about physical activity and diet	UK	To explore the barriers to initiating and maintaining regular physical activity (PA) among UK Indian, Pakistani and White British adults in their 60s	Focus groups and interviews	Number (% male), 127 (31%). Mean age (range) in years, 65 (60–70). Ethnic categories: South Asian (Indian (*n* = 13) and Pakistani (*n* = 33)) and White (*n* = 81)
	Barriers and facilitators to living a healthier lifestyle				
Penn et al. [59]	Current behaviours, knowledge and attitudes about physical activity and diet	Middlesbrough, England	To investigate Pakistani female participants’ perspectives of their behaviour change and of salient intervention features	Interviews	Number (% male), 20 (all women). Mean age (range) in years, 34 (26–45). Ethnic categories: South Asian (Pakistani)
	Barriers and facilitators to living a healthier lifestyle				

### Knowledge and Attitudes About Diabetes Risk

In total, we identified 11 studies that reported information on the knowledge and attitudes about diabetes risk among ethnic minority populations [38, 41–43, 50, 52, 53, 55, 57, 59, 63]. All of these 11 studies reported on South Asian minority ethnic populations.

In Bangladeshi [41, 42] and combined South Asian communities [57], knowledge about T2DM was reported to be high. People from Bangladeshi, Pakistani and Indian communities were also aware of their increased risk of developing T2DM [41, 59, 63], owing partly to exposure to the condition through family members [41, 63], which could motivate lifestyle choices [41, 59]. It was recognised that T2DM was partially preventable by avoiding certain foods, such as sugar and saturated fat [41, 42, 57]. In contrast, other studies reported that participants did not know which aspects of lifestyle behaviours or, indeed, whether obesity could contribute to the development of T2DM and cardiovascular disease [38, 50, 55].

Perceived lack of individual control in developing T2DM was prevalent among the South Asian communities studied. External causes such as genetics [41, 43, 52] and stressful life events, often exacerbated by immigration [38, 41, 50, 53, 55, 57], were perceived to be important. Studies also noted fatalistic health beliefs in these communities [41, 55, 57, 63] although some attributed these to older generations [41], acknowledging that individuals were responsible for protecting their own health [38, 41].

### Current Behaviours, Knowledge and Attitudes About Physical Activity and Diet

We identified 25 studies that reported on current behaviours, knowledge and attitudes about physical activity and diet in minority ethnic populations [9, 35, 37–39, 41–45, 47, 48, 50, 52, 54–57, 61–67]. Seventeen of these reported physical activity behaviours [9, 37–39, 41–43, 45, 47, 50, 52, 54, 55, 57, 62, 64, 67] and 11 reported dietary behaviours [9, 35, 38, 44, 48, 55, 56, 61, 63, 65, 66].

The majority of the studies (*n* = 20) reported data on South Asian minority ethnic groups (Indian, Pakistani and Bangladeshi) [35, 37–39, 41–45, 47, 50, 52, 55, 57, 62–67]. Four of the studies additionally reported data on Black African, Black Caribbean and Chinese individuals [9, 48, 54, 61]. The remaining study was restricted to the British Somali population [56].

#### Physical Activity

A key population-based study showed differences in adherence to physical activity recommendations by minority ethnic group [9]. The survey reported results from the Health Survey for England 2004 and observed higher rates of adherence in Irish and Black Caribbean men (39% and 37%, respectively) and Black Caribbean, Black African and Irish women (31%, 29% and 29%, respectively). All South Asian groups appeared to do less physical activity than the White population. In another survey, lower levels of sports participation were observed among ethnic minority groups as a whole, compared with the White British population. However, stratification by ethnic group revealed higher participation in ‘mixed’ and ‘Chinese and other’ ethnic minority populations [54]. Similarly, systematic review evidence also suggests that South Asian minority ethnic populations, in particular South Asian women and older individuals, have lower levels of physical activity compared with White British populations [39]. More recent studies suggest that this pattern is persisting [64, 67].

Studies of South Asian participants showed that whilst they were generally aware of the health benefits associated with physical activity [38, 41, 47, 48, 57], they had more limited understanding of the actual levels of physical activity required to gain health benefits [37, 38, 41, 48, 50]. Five studies reported cultural differences in relation to perceptions of physical activity as an ‘organised’ activity; housework and namaz (prayer), for example, were seen as sufficient to gain health benefits [41, 43, 45, 52, 57]. Other studies also reported general resistance and lack of motivation to carrying out any organised physical activity that involved breathlessness, increased activity or sweating [41, 42, 50, 55, 57]. Information needs were also evident; South Asian women with T2DM felt that they needed more guidance from healthcare professionals on appropriate and safe levels of physical activity [62].

#### Diet

Two national surveys in England and Scotland collected information on dietary intake among different ethnic populations. In England, respondents from all minority ethnic groups were more likely to report meeting the fruit and vegetable intake (‘5 as day’) recommendations than the White population [9]. However, in Scotland, only Chinese and African-Caribbean respondents were more likely to report meeting these recommendations than the White population and South Asian groups were less likely [61]. Similarly, a study in the UK Somali population suggested a lower consumption of fruit and vegetables [56].

Eating patterns in South Asian communities vary substantially by generation, household, region and country. Traditional South Asian diets that are low in meat, fish and dairy products and high in chapatis or rice, pulses, fruit and vegetables are also high in fibre and low in fat [65, 66]. However, dietary transition has been observed after migration whereby consumption of convenience foods increases and vegetable consumption decreases, leading to a less healthy diet [35, 44].

Research on African-Caribbean, Indian, Pakistani and Bangladeshi minority ethnic groups has shown that the majority had a good understanding of healthy eating messages but relatively poor understanding of foods that were high in saturated fat and fibre [48]. Among South Asians, knowledge of high saturated content and sugar of traditional South Asian food varied [48, 55, 63]. Some minority ethnic groups perceived that traditional diets were healthier than Western diets [38, 48, 55]. Somali participants made a cultural association between fruit and vegetables and poverty, and red meat with affluence, which impeded their understanding of a healthy diet [56]. Similarly, some studies indicated a lack of knowledge about how to prepare healthy food in people with and without T2DM [38, 56, 63].

### Barriers and Facilitators to Living a Healthier Lifestyle

Table 2 summarises the literature on barriers and facilitators to healthy lifestyles. We identified 21 relevant studies that reported on the barriers and facilitators to living a healthier lifestyle among ethnic minority populations [36–38, 40–43, 45–47, 49–52, 55, 57–60, 62, 63]. Seven included participants with T2DM [40, 42, 51, 52, 58, 62, 63] and 14 without T2DM [36–38, 41, 43, 45–47, 49, 50, 55, 57, 59, 60].

**Table 2:** Summary of barriers and facilitators to living a healthy lifestyle

Barriers	Supporting literature	Facilitators	Supporting literature
Social norms and values			
Prioritisation of work over physical activity to provide for the family	Carroll et al. [37], Horne et al. [45], Jepson et al. [47], Lawton et al. [52], Netto, McCloughan, and Bhatnagar [57] and Sriskantharajah and Kai [62]	Concern that weight gain might compromise role of family carer/wage earner	Ludwig et al. [55]
Cultural expectation that women remain at home/do not exercise outside	Carroll et al. [37], Farooqi et al. [38], Grace et al. [41], Khanam and Costarelli [50] and Lawton et al. [52]	Diagnosis of type 2 diabetes	Fleming et al. [40]
Cultural importance of serving traditional food and expectations of family/social circle	Bush et al. [36], Farooqi et al. [38], Grace et al. [41], Greenhalgh et al. [43], Jamal [46], Lawton et al. [51], Ludwig et al. [55], Netto et al. [57] and Penn et al. [59]	Desire to lose weight/keep healthy	Farooqi et al. [38], Netto et al. [57] and Penn et al. [59]
Need to adhere to cultural expectations of food and eating practises to avoid being alienated	Fleming et al. [40], Lawton et al. [52] and Stone et al. [63]	Obesity associated with being unattractive, unhealthy and low fertility	Greenhalgh et al. [42]
Different perception of body image, body weight and social acceptability	Grace et al. [41], Johnson [49], Khanam and Costarelli [50], Ludwig et al. [55], Netto et al. [57] and Patel et al. [58]		
Structural factors			
Uncertainty as to the appropriateness of exercise clothing	Grace et al. [41]	Education perceived to empower women to resist social pressure and go out to exercise	Grace et al. [41]
Conflict between music/images in the gym and cultural beliefs	Khanam and Costarelli [50]	Incorporation of physical activity classes in places of worship	Jepson et al. [47]
Language barriers	Carroll et al. [37], Khanam and Costarelli [50], Lawton et al. 2006 [52], Sriskantharajah and Kai [62] and Stone et al. [63]		
Fear for personal safety	Greenhalgh et al. [43] and Rai and Finch [60]		
Fear of racial harassment and abuse	Greenhalgh et al. [43] and Johnson [49]		
Fear of travelling outside immediate community	Grace et al. [41]		

All of the studies focused on South Asian minority ethnic groups and most reported on their perspectives as one group [36–38, 45, 47, 49, 51, 52, 57, 58, 60, 62, 63]. The remaining eight studies sought the perspectives of Bangladeshi [41–43, 50], Pakistani [46, 55, 59] or Indian [40] communities separately. Seven of the studies explored the views of women only [36, 37, 50, 55, 58, 59, 62].

#### Social Norms and Values

A common theme of the studies was the need to financially care and provide for family members and thus prioritise work over physical activity [37, 45, 47, 57]. However, concern that weight gain could compromise the role of family carer or wage earner sometimes motivated physical activity [55]. Similar barriers were observed among South Asians with T2DM [52, 62].

Gender norms were also found to impede opportunities for South Asian women to engage in physical activity. There were cultural expectations to remain in the home, regardless of T2DM status [41, 50] and potential disapproval from other community members if seen walking or exercising outside [37, 38, 52]. Muslim women who exercised in facilities with other men anticipated disappointment from male family members [59], but there was a suggestion that ‘educated’ Muslim women were more empowered to resist social pressure [41].

Resistance to change was also observed with regard to cooking practises. Reducing the amount of ghee or oil was seen to render the food tasteless and could even be shameful [38, 41, 55, 57]. The cultural importance of serving traditional food [57] and expectations of family members and the wider social circle also prevented dietary changes [36, 38, 43, 46, 51, 57, 59]. In a study of Gujarati Muslim men, family members recognised that a participant’s diagnosis of T2DM necessitated changes to his diet, but not to their own [40]. Despite this, several studies found that some South Asian women were making healthier changes to their diets, such as eating smaller portions and reducing fat [38, 57, 59].

Another barrier related to cultural pressure when visiting family members’ homes or attending celebratory events. Feelings of having to live up to cultural expectations of food and eating practises to avoid being alienated continued, even after being diagnosed with T2DM [40, 52, 63].

Perceptions of body weight, body image and social acceptability of being overweight have been shown to differ between minority ethnic groups [41, 49, 50, 55, 57]. In one study, health professionals asserted that Bangladeshi people associated obesity with good health and fertility [41]. Conversely, another study of Bangladeshi participants with T2DM found that they were able to identify accurately if they were overweight and perceived obesity to be unattractive, unhealthy and associated with low fertility [42]. In a study comparing perceptions of weight among South Asian and White British women with T2DM, South Asian women were more likely to perceive their body weight as normal, despite being overweight [58].

#### Structural Factors

Several studies found cultural barriers to participation in physical activity related to mixed gender venues [38, 41, 45, 52, 62] and facilities [37, 38, 52, 59] among Muslim male and female participants. Muslim women also expressed uncertainty as to the appropriateness of wearing traditional clothes to exercise or western clothes which could draw attention to their bodies [41]. In one study, British Bangladeshi participants felt that the music and images to which they were exposed in the gym conflicted with their cultural beliefs [50]. Some of these barriers could be overcome by incorporating physical activity classes in places of worship (mosques) [47].

Language barriers were also found to discourage participants from taking part in physical activity because they would be unable to ask for help [52, 62] or understand instruction [50]. Some participants relied on relatives to accompany them and act as translators [37, 63]. Such barriers might be expected to lessen in importance over time in the UK owing to the decrease in the proportion of first-generation migrants in the South Asian population.

An additional structural barrier related to concerns over personal safety [43, 60], racial harassment and abuse [43, 49], which deterred participants from using facilities and open spaces in the community. Additional fears were expressed in relation to travelling outside the perceived safety of the immediate community [41].

## Discussion

In this narrative review, we have synthesised the literature in relation to barriers and facilitators to healthy lifestyle changes in minority ethnic populations in the UK. We have also summarised the literature on knowledge and attitudes about diabetes risk, physical activity and diet in ethnic minority populations.

The first point to note from the findings in this review is that most of the literature related to South Asian communities. This is not entirely surprising given that this population is the most widely represented ethnic minority group in the UK and that T2DM is known to be more prevalent in this population [68–70]. However, the dearth of literature on other ethnic minority populations living in the UK is a concern, and we would recommend further research in these communities.

Secondly, it is important to recognise that ethnic minority populations are not a homogeneous group, as is reflected in the diverse and often contrasting findings from the studies included in the review. Ethnicity is defined by a complex interplay of characteristics, which include spoken language, religious beliefs and common heritage, and people within and between ethnic groups vary considerably. Therefore, when making observations, it is important to consider contextual as well as cultural barriers, and caution needs to be applied in assuming our findings are transferable. Despite this, we can make some general observations from the findings to consider when developing culturally appropriate lifestyle interventions, which are particularly relevant to South Asian populations on whom most of the research was focused.

With regard to knowledge and attitudes about diabetes risk, most of the South Asian participants in our studies recognised that they were at an increased risk of developing T2DM. However, many did not attribute this increased risk to lifestyle behaviours or obesity and often perceived external events, such as genetics, stress and fatalistic beliefs, to be more important. These findings are largely supported in the USA where South Asian-Indian participants recognised their increased cardiovascular risk but were generally sceptical about the role of obesity, citing destiny or ‘karma’ as more likely influences [71].

We found some evidence that South Asian minority populations were less likely to engage in physical activity compared with the White population and that South Asian participants were unsure how much physical activity was needed to give health benefits. It was not clear whether or how dietary intake varied between South Asian and White populations, but there were some misunderstandings, some of them cultural, about foods that constituted a healthy diet.

In terms of barriers and facilitators to living a healthy lifestyle, family and community pressures to conform to the social norms and values in South Asian cultures were seen to be particularly important. Barriers included prioritising work over physical activity to provide for the family, the need to serve and eat traditional food and different perceptions of a healthy body weight. Similar findings have been found in South Asian Indians living in the USA [72] and Australia [73] where family responsibilities were prioritised over physical activity. Interestingly, in both of these studies, car travel was seen as a barrier to physical activity, which was not mentioned in any of the studies included in our review. Other barriers to physical activity included fear of racial harassment or abuse when exercising and, for women, expectations to remain in the home, fear for personal safety, lack of same gender venues and concerns over the acceptability of wearing ‘western’ exercise clothing. Facilitators included concern that weight gain might compromise family/carer responsibilities, desire to be healthy, T2DM diagnosis, and exercise classes held in ‘safe’ environments such as places of worship.

### Strengths and Weaknesses

The purpose of this narrative review was to summarise the evidence by giving an overview of primary research published in this topic area. We did not carry out a systematic search of the literature nor include grey literature. We also did not appraise the quality of the studies. It is recognised that narrative reviews are prone to selection bias [74] and provide weak evidence for making clinical decisions about the care of individual patients [75]. However, we are able to present a broad perspective on barriers and facilitators to healthy lifestyle changes in minority ethnic, in particular South Asian, populations.

### Closing Remarks

Minority ethnic populations experience a disproportionate burden of health inequalities compared with the White population, including an increased risk of T2DM. Findings from this review highlight the importance of considering social, structural and cultural contexts when engaging South Asian populations in T2DM preventive strategies. Further research of other ethnic minority populations is urgently needed to explore knowledge and attitudes about diabetes risk and lifestyle factors and to identify barriers and facilitators to healthy lifestyle changes.
